# Humid tropical rain forest has expanded into eucalypt forest and savanna over the last 50 years

**DOI:** 10.1002/ece3.70

**Published:** 2012-01

**Authors:** David Y P Tng, Brett P Murphy, Ellen Weber, Gregor Sanders, Grant J Williamson, Jeanette Kemp, David M J S Bowman

**Affiliations:** 1 School of Plant Science, University of TasmaniaHobart, TAS 7005, Australia; 2 Geographic Information Science Center of Excellence, South Dakota State UniversityBrookings, South Dakota; 3 Wet Tropics Management AuthorityCairns, QLD 4870, Australia; 4 Queensland HerbariumTownsville, QLD 4810, Australia

**Keywords:** Atmospheric CO_2_, biome shifts, boundary dynamics, global drivers, rain forest expansion, Wet Tropics World Heritage Area

## Abstract

Tropical rain forest expansion and savanna woody vegetation thickening appear to be a global trend, but there remains uncertainty about whether there is a common set of global drivers. Using geographic information techniques, we analyzed aerial photography of five areas in the humid tropics of northeastern Queensland, Australia, taken in the 1950s and 2008, to determine if changes in rain forest extent match those reported for the Australian monsoon tropics using similar techniques. Mapping of the 1950s aerial photography showed that of the combined study area (64,430 ha), 63% was classified as eucalypt forests/woodland and 37% as rain forest. Our mapping revealed that although most boundaries remained stable, there was a net increase of 732 ha of the original rain forest area over the study period, and negligible conversion of rain forest to eucalypt forest/woodland. Statistical modeling, controlling for spatial autocorrelation, indicated distance from preexisting rain forest as the strongest determinant of rain forest expansion. Margin extension had a mean rate across the five sites of 0.6 m per decade. Expansion was greater in tall open forest types but also occurred in shorter, more flammable woodland vegetation types. No correlations were detected with other local variables (aspect, elevation, geology, topography, drainage). Using a geographically weighted mean rate of rain forest margin extension across the whole region, we predict that over 25% of tall open forest (a forest type of high conservation significance) would still remain after 2000 years of rain forest expansion. This slow replacement is due to the convoluted nature of the rain forest boundary and the irregular shape of the tall open forest patches. Our analyses point to the increased concentration of atmospheric CO_2_ as the most likely global driver of indiscriminate rain forest expansion occurring in northeastern Australia, by increasing tree growth and thereby overriding the effects of fire disturbance.

## Introduction

Determining the dynamics of tropical rain forest and savanna boundaries is a prerequisite for a comprehensive understanding of a major feedback system within the global carbon cycle, as these two geographically and ecologically linked biomes constitute substantial above- and belowground carbon stocks and fluxes on a global scale. Brazilian rain forests, for instance, store around 250–300 t C ha^–1^ and the adjacent tropical savanna stores 135 t C ha^–1^ (Behling 2002). Numerous reports on the expansion of rain forest (Puyravaud et al. 1994, 2003; Schwartz et al. 1996; Happi 1997; Delègue et al. 2001; Banfai and Bowman 2006; Banfai et al. 2007; Silva et al. 2008) and increasing biomass in both rainforest (Lewis et al. 2009) and savanna worldwide (Bowman et al. 2001; Briggs et al. 2005; Lehmann et al. 2008; Wigley et al. 2010) signal that these biomes are potentially important global carbon sinks. The physiological mechanisms causing these sinks are related to more efficient nutrient and water use by trees in response to increased atmospheric CO_2_ concentrations (Drake et al. 1997; Poorter 1998). Increases in atmospheric CO_2_ concentrations have also been correlated to increased growth rates of trees (Bond and Midgley 2000), possibly contributing to the expansion of forests (Bond et al. 2003; Behling et al. 2005).

However, whether rain forest expansion or general vegetation thickening is driven by local or global drivers is a contentious issue. Many studies show that local factors (e.g., fire regimes, geology, topography) can play an important role in rain forest expansion or woody vegetation increases (Archer et al. 1995; Bond et al. 2003; Russell–Smith et al. 2004b). A difficulty in such studies lies in disentangling the importance of global drivers from the “noise” of local variation (Wigley et al. 2010). Moreover, many such landscape-scale studies (Russell–Smith et al. 2004b; Banfai and Bowman 2006) also suffer from the confounding effects of spatial autocorrelation (Murphy et al. 2010).

Advances in geospatial techniques in the past decade have enabled the study of tropical rain forest systems at a landscape scale, and the use of geographic information systems (GIS) are increasingly valuable in ecological studies of vegetation dynamics (e.g., Banfai and Bowman 2005; Brook and Bowman 2006; Wigley et al. 2010). Indeed, Bowman et al. (2010) have summarized a range of aerial photographic studies undertaken in the Australian monsoon tropics that disclose a regional increase in forest cover, despite fire regimes that are damaging components of the region's savanna biodiversity.

Here, we determine the rates of landscape change and landscape conditions associated with rain forest expansion in the humid tropics of Australia to see if there is a trend similar to that in the Australian monsoon tropics. We assess change in rain forest boundary locations in a 644.3-km^2^ study area within in the Wet Tropics World Heritage Area of northeastern Queensland using aerial photography taken in the 1950s (1951–1955) and 2008. We use geospatial statistics to determine to what extent rates of rain forest change were mediated by environmental conditions (geology, elevation, topographic position, slope, aspect). Using our estimated rates of change in rain forest extent, we also project the effects of expanding rain forests on the spatial extent of other vegetation types. We expected that if global drivers were also driving rain forest expansion in the Australian humid tropics, the expansion would occur indiscriminately across all environmental conditions.

## Materials and methods

### Study area

**Figure 1:**
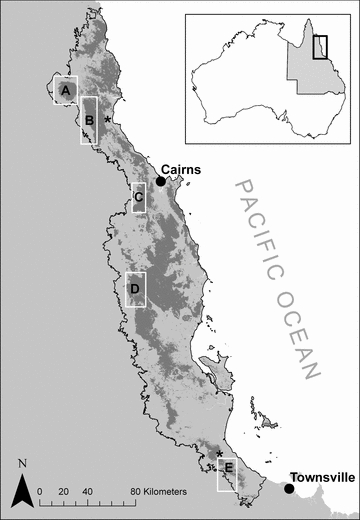
Selected sites for mapping of rain forest change, denoted by rectangles representing: Mt. Windsor (A), Mt. Carbine (B), Clohesy (C), Koombooloomba (D), and Paluma (E). Shaded areas denote the mapped extent of rain forest and Wet Tropics Bioregion is represented by a black outline. Weather stations used in the current study are indicated with asterisks, the northern-most being the Mossman Central Mill weather station and the southern-most being the Paluma Ivy Cottage weather station. The inset shows the outline of Australia with the state of Queensland shaded and the bounded rectangle denotes the whole study area.

The study area was situated in the Wet Tropics Bioregion, a humid tropical zone in northeastern Queensland, Australia (Fig. 1), covering approximately 1.8 million hectares. The area is characterized by a mosaic of naturally and artificially fragmented areas of tropical rain forest interspersed with fire-prone vegetation (e.g., grassland, open eucalypt woodland, and forest [Hopkins et al. 1993; Hilbert et al. 2001]) and pasture and agricultural fields. Rain forest in this region is physionomically and floristically diverse, ranging from species-rich, complex vine forest developed on relatively nutrient-rich, moist but well-drained soils, to structurally simple rain forest types on oligotrophic moist soils (Webb 1959; Webb and Tracey 1981). Prior to European settlement in the 19th and 20th centuries, rain forest covered an estimated 965,000 ha. Subsequent human impacts resulted in a reduction in the area of rain forest to approximately 750,000 ha. Some small areas of rain forest are privately owned, although most of the remaining rain forest areas in northeastern Queensland were secured by the declaration of the Wet Tropics World Heritage Area in 1988 (Lane and McDonald 2000).

A distinctive feature of uncleared rain forest tracts in the Queensland humid tropics is the occurrence of physiognomically abrupt boundaries between rain forest and eucalypt-dominated vegetation (Unwin 1989; Harrington and Sanderson 1994). A tall open forest formation dominated by tall (>40 m) eucalypts (e.g., *Eucalyptus grandis* and *E. resinifera*) typically forms a narrow fringe ranging in width from around 300 m to 4 km along the rain forest margins on the western side of the Wet Tropics Bioregion (Harrington and Sanderson 1994; Harrington et al. 2000). It has been suggested that this forest formation is in danger of being replaced by expanding rain forest (Harrington and Sanderson 1994; Goosem et al. 1999) making some elements of the biodiversity vulnerable to local extinction. The remainder of the terrestrial vegetation comprises a variable mosaic of low to medium height eucalypt-dominated open forests and woodlands occupying a broad range of freely draining substrates, heaths restricted to shallow, infertile soils, and *Acacia*, *Lophostemon*, or *Syncarpia* dominated forests.

The sites selected for the current study lie within the upland regions of Mt. Windsor, Mt. Carbine, Clohesy, Koombooloomba, and Paluma (Fig. 1; Table 1). Vegetation in the five study sites comprises a representative subset of the uncleared vegetation mosaics found within the Wet Tropics Bioregion, which includes rain forest and a range of vegetation types on drier areas. The geology of the five areas was highly variable, but granite and mudstone made up the bulk of the sites, with localized areas of basalt, that has been the predominant target of land clearing (Table 1).

**Table 1:** Details of study sites

Study sites	Date(s) of first time period aerial photography	*Area (ha)	Altitude range (m.a.s.l.)	Geology
Mt. Windsor	1951–1955	12,137	300–1328	Paleozoic granite batholiths, Hodgkinson formation, metamorphics and Devonian mudstone
Mt. Carbine	1955	14,882	311–1348	Paleozoic granite batholiths, Hodgkinson formation, metamorphics and Devonian mudstone
Clohesy	1949	11,003	350–1310	Lower Permian granites and Devonian mudstone and metamorphic
Koombooloomba	1951	18,175	672–1182	Carboniferous acid volcanics, Late tertiary basalts, Middle carboniferous granite complexes, and Quarternary Colluvium and Alluvium
Paluma	1950	8232	240–1003	Lower Permian to middle carboniferous granites and middle Paleozoic metamorphics

* The total area of grid cells for each site used in the final analysis.

Mean annual rainfall over the study period at the Mossman Central Mill weather station (Fig. 1) exceeded 2300 mm, mostly falling between December and April, with the highest rainfall occurring in January and the lowest in July. Mean annual rainfall at the Tully Sugar Mill weather station (Fig. 1) exceeded 4100 mm, mostly falling between December and April, with the highest rainfall occurring in January and the lowest in August (Australian Bureau of Meteorology 2010).

### Mapping rain forest change

Available 1950s black and white aerial photos (scales ranging from 1:24,000 to 1:30,000) for the five areas (Fig. 1; Table 1) were scanned at 1690 dots per inch, orthorectified and stitched to create an orthomosaic. A 30-m horizontal resolution digital elevation model (DEM) (Shuttle Radar Topographic Mission Level 2 data, licensed for use by Geoscience Australia) provided the rectification surface. A color 2008 orthomosaic covering the entire Wet Tropics Bioregion was used as a comparison image for vegetation change, and provided a 0.5-m resolution control layer for spatial referencing and adjustment of the 1950s photography. Features such as drainage lines, rocky outcrops, buildings, and occasionally the center point of a single tree canopy were aligned to corresponding features in the 2008 orthomosaic.

To estimate temporal change, we employed a grid approach and layered 50 m × 50 m cells over each of the five areas for both time periods and attributed each cell for vegetation type. Collectively, all five grid areas encompassed an area of 644.3 km^2^. These grid cells were positioned to include both rain forest and eucalypt forest/woodland vegetation across vegetation boundaries. The vegetation for each grid cell was attributed by assigning a status of being either rain forest or “savanna” (defined here as eucalypt forest and other open woodland types), based on canopy openness (closed canopy = rain forest; open canopy = savanna) and discernable understorey components. Individual eucalypts (genera *Eucalyptus* and *Corymbia*), *Lophostemon* and *Syncarpia* can readily be recognized on aerial photographs by a fuzzy canopy, or by the general color in the 2008 orthomosaic. To facilitate the process of vegetation attribution from nonstereo image interpretation, and as an added measure of accuracy, we overlaid a 2008 vegetation map provided by the Wet Tropics Management Authority as an additional guideline for determining vegetation type. Grid cells in which both rain forest and savanna occurred were attributed based on the dominant vegetation type. Grid cell areas that covered bare rock, roads, water bodies, built-up areas, or plantations were excluded from the subsequent analysis.

To determine the linear distance of vegetation change, we selected, for each of the five sites, 100 points on the 1950s rain forest boundary. For each of the 500 points, the distance to the nearest 2008 rainforest boundary was measured.

### Correlates of rain forest change

The grid cells used for attributing vegetation type were also attributed for environmental variables including elevation, geology, proximity to water bodies or drainage systems, and distance to rain forest (Table 2). We excluded rainfall as it was strongly correlated with elevation. Elevation was calculated from the same DEM used for aerial photo rectification. A topographic position index (TPI; Jenness 2005) was calculated from the same DEM, using a search radius of 500 m. TPI provides a measure of the difference in elevation of a location and the mean elevation of the surrounding area, and is therefore useful for classifying locations as ridges, valleys, etc. The distance from preexisting rain forest was extrapolated from the grid cells attributed for rain forest in the 1950s.

**Table 2:** Local environmental correlates deemed to have an influence on rain forest change

Variable	Description	Hypothesized effect
Aspect	Aspect was incorporated as a composite variable consisting of “northness” [cosine(aspect) ×%slope] and “eastness” [sine(aspect) ×%slope]. Thus, “northness” and “eastness” were indices ranging from –1 (steep south or west-facing slope) to 1 (steep north or east-facing slope).	Lower probability of expansion on steeper slopes due to increased fire intensity and reduced moisture trapping, and greater probability of expansion on steeper slopes correlated with topographic protection.
Distance from preexisting rain forest	Distance (m) from the nearest rain forest patch margin as mapped in the earlier time period (1950s) from the five sites using the first time period orthomosaic.	Declining probability of invasion at points distant from preexisting rain forest due to limitations on seed dispersal.
Elevation	Elevation (m) above sea level from 30-m resolution DEM.	Greater probability of expansion at higher elevations due higher rainfall and lower evaporation rates.
Geology	Broad classes extracted from Australian Geological Survey 1:250,000 map for the region.	Expansion rates will vary with geology due to differences in fertility and water-holding capacity.
Slope	In degrees, calculated from a 30-m digital elevation model (DEM).	Lower probability of expansion on steeper slopes, due to higher fire intensity, greater water run off.
TPI	Topographic Position Index (Jenness 2005) determined for each grid cell of a 30-m DEM by calculating the difference between the elevation of the grid cell and the mean elevation calculated from all grid cells in a circular window of radius 500 m centered on the cell of interest.	Lower probability of expansion on ridges, due to higher fire activity and lower water availability.
Distance to drainage systems/water bodies	Proximity (m) to water bodies or drainage systems.	Greater probability of expansion close to water due to higher water availability, fire protection, and propagule dispersal in water.

### Modeling rain forest change

We treated our response variable as binary (i.e., 0 = savanna remained savanna; 1 = savanna changed to rain forest). Models representing all combinations, without interactions, of the seven environmental correlates (Table 2) considered to be relevant to rain forest change were constructed as generalized autoregressive error models (GAR_err_), using a binomial error family with logit link. This type of model was recently developed by Murphy et al. (2010) to analyze spatially autocorrelated nonnormal data. It is similar to the simultaneous autoregressive error model for normal data (Cressie 1993) but can cope with nonnormal data types such as a generalized linear model. This type of spatial model is limited to 4000 observations, so we chose a random sample of our total dataset. Because virtually no conversion from savanna to rain forest occurred >1 km from a rain forest boundary, we selected 4000 points from within this distance. We confirmed that the GAR_err_ models successfully accounted for residual spatial autocorrelation using correlograms based on Moran's I (Dormann et al. 2007).

Models were evaluated using the Bayesian Information Criterion (BIC), a model selection index favoring both model fit and model simplicity (Burnham and Anderson 2002). BIC is analogous to the more widely used Akaike Information Criterion (AIC), but tends to penalize complex models more heavily than AIC. Hence, it tends to be more appropriate for large datasets where the main underlying drivers are of primary interest (Link and Barker 2006). Lower values of BIC indicate greater support for a model, relative to other models in the same candidate set. From BIC, evidence weights (*w_i_*) were calculated for each model and these are equivalent to the probability of a given model being the best in the candidate set. The importance of each variable was evaluated by calculating *w*+, the sum of *w_i_* for all models in which that variable occurred. For each variable, *w*+ is equivalent to the probability of the best model containing that variable, and is a useful expression of the weight of evidence for the importance of the variable. We considered that *w*+ values of < 0.73 were indicative of substantial model selection uncertainty, and that a relationship between the response and the explanatory variable in question was not well supported by the data. A *w*+ value of 0.73 is equivalent to a BIC difference of two units between the models containing the variable under examination and those not containing it. A difference of two units is a common “rule of thumb” used in ecological studies to assess evidence of an effect (Richards 2005).

We also performed a post hoc test to determine the effect of vegetation type (i.e., tall eucalypt forest vs. dry eucalypt forest: Queensland Herbarium 2009) on the probability of conversion to rain forest. Using BIC, we compared the best model from the a priori candidate set, with the same model incorporating a term representing vegetation type.

### Projected rain forest expansion into tall open forest

Using standard GIS functions, a 100-m square lattice of points was generated across the entire extent of tall open forest in the Wet Tropics Bioregion, as per Queensland Regional Ecosystems vegetation mapping (Queensland Herbarium 2009), and the distance from preexisting rain forest was calculated for each point. Using a geographically weighted estimate of the rate of boundary expansion from the five study sites, we estimated the proportion of tall open forest remaining over various time periods up to 2000 years. Using a weighted average of the linear boundary change allows for a more realistic analysis, as it takes into account the variation in mean linear boundary change across the five study sites. We considered this modeling exercise conservative and representative of a “worst-case-scenario” in terms of tall eucalypt forest loss, as it assumes: (1) there will be no landscape scale perturbances at the rain forest margins (e.g., droughts, natural fires, or cyclonic damage) that might affect the rate of rain forest expansion, (2) rain forest expansion will advance across the landscape unchecked by preexisting geographical or climatic barriers, and (iii) tall open forest eucalypt woodland boundaries are static and tall eucalypt forest does not advance ahead of the advancing rain forest.

## Results

### Changes in rain forest area and linear spread

**Figure 2:**
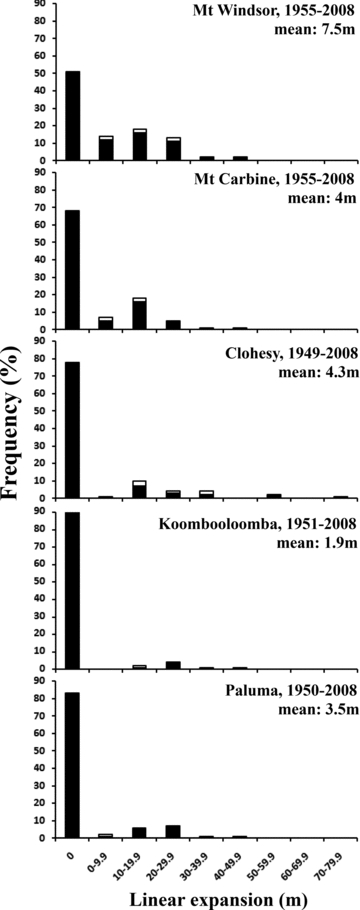
Linear expansion of the rain forest margins on the five study sites as measured from 100 random paired points from each site. Black bars denote points where the rain forest boundary had expanded in 2008; and white bars denote points where the savanna boundary has expanded. The mean decadal linear rain forest expansion (m) over the study period is indicated for each site.

At all five study sites our results show that most boundaries remained stable, but where change occurred, rain forest expanded into surrounding savanna (Fig. 2; Table 3) with a net rain forest expansion of 732 ha. The extent of rain forest expansion was greatest at Mt. Windsor (8.5%) and least at Paluma (0.8%). Conversion of rainforest to savanna was negligible.

**Table 3:** Change in area extent of rain forest and savanna in the five study sites from the 1950s to 2008

	Year	Rain forest area (ha)	Savanna area (ha)	Proportional change (Rain forest to Savanna) (%)	Proportional change (Savanna to Rain forest) (%)	Net change in rain forest area (%)
Mt. Windsor	1950s	4356	7781	0.8	5.2	8.5
	2008	4724	7413			
Mt. Carbine	1955	5232	9650	0.8	1.3	1.6
	2008	5313	9569			
Clohesy	1949	5075	5929	0.1	1.6	1.8
	2008	5165	5839			
Koombooloomba	1951	5159	13,016	2.6	2.3	3.1
	2008	5319	12,856			
Paluma	1950	4224	4008	0	0.8	0.8
	2008	4257	3975			
Combined	1950s	24,046	40,384	0.9	2.2	3.2
	2008	24,778	39,652			

In terms of linear boundary shifts, 25% of the 500 paired sampling points across the five sites exhibited change in the location of rain forest boundaries (Fig. 2). Across all sites, most of the boundaries showed that rain forest expansion was less than 30 m since the 1950s (Fig. 2), and at an average rate of 0.6 m per decade. Comparatively, savanna expansion was very limited (Fig. 2).

### Correlates of change

**Figure 3:**
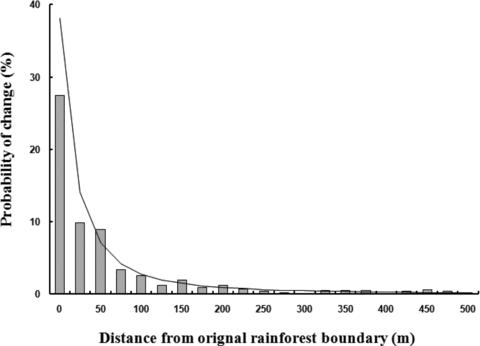
The observed (bars) and predicted (line) probabilities of conversion of savanna to rain forest in relation to distance to the original rain forest boundary. The model predictions are based on multimodel averaging of the entire candidate set of models, weighted according to *w_i_* and assuming mean values for all other variables.

There was a very strong effect of distance from the original rain forest boundary on the probability of conversion of savanna to rain forest. The probability (*w*+) of “distance to rain forest” appearing in the best model of savanna conversion was >0.99 (Fig. 3; Table 4). Little savanna situated more than 200 m from a rain forest boundary became rain forest. No other variables had any clear effect on the probability of conversion from savanna to rain forest (Table 4). Out of 256 models generated, the best model of savanna conversion (*w_i_* = 0.91) explained 32% of the residual deviance at the five sites combined.

**Table 4:** Importance values (*w*+) of environmental predictors of combined rain forest expansion at the five study sites, based on the Bayesian Information Criterion (BIC). “*w*+” can be interpreted as the probability of that variable being in the best model. As a “rule of thumb,” values of *w*+≥0.73 (shown in bold) can be interpreted as clear evidence of an effect (Richards 2005)

Variable	*w*+
Distance to preexisting rain forest	**>0.99**
Topographic position index	0.04
Elevation	0.02
Slope	0.02
Geology	0
Distance to drainage	0.02
Aspect	0

**Figure 4:**
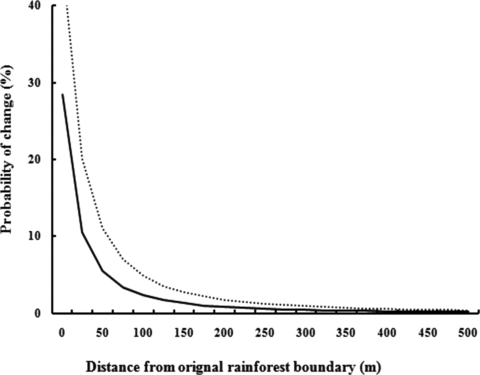
The probabilities of conversion of tall open forest (dashed line) and other dry forest types (unbroken line) into rain forest in relation to distance to the original rain forest boundary.

Our analyses on the effect of vegetation type on the probability of rain forest expansion show that rain forest was more likely to expand into adjacent tall open forest than into other woodland types. The difference between rain forest expansion into tall open forest and other woodland types was significant (ΔBIC > 2), although the magnitude of the difference was not large (Fig. 4).

### Projected rain forest expansion into tall open forest

**Figure 5:**
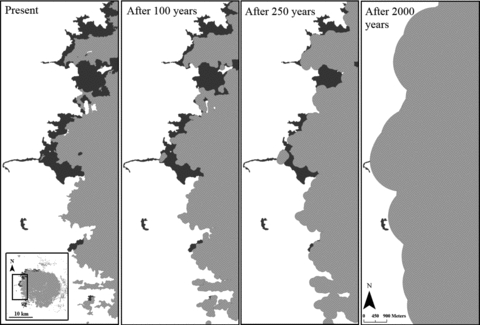
Projection of the engulfment of tall open forest (dark gray) by rain forest (light gray) at Mt. Windsor after 100, 250, and 2000 years, based on an average rain forest expansion rate of 6 m decade^–1^. Inset shows the Mt. Windsor study site (Fig. 1) and the bounded area is the selected area for illustrating the time series. Note how the irregular shape of the tall open forest patches slows the rate of rain forest engulfment.

**Figure 6:**
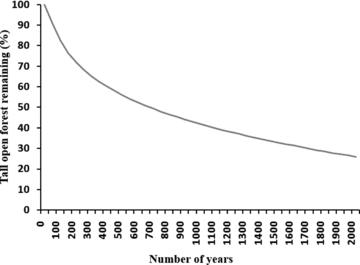
The extent of tall open forest predicted to be engulfed by rain forest over time.

We project that after 100 years of rain forest expansion, there would be over 85% of tall open forest area remaining (Figs. 5 and 6). The sharpest decrease in tall open forest extent is predicted to occur within the first 250 years, during which 30% of tall open forest area would be engulfed by rain forest. Expansion of rain forest into tall open forest is predicted to slow after the first 250 years, and after 2000 years there is still more than 25% of the original area of tall open forest remaining (Fig. 6).

## Discussion

Although most rainforest boundaries in our study were stable over a 60-year period, those that did change all expanded into surrounding eucalypt forest and savanna resulting in a regional increase in rain forest. Such rain forest expansion has been documented in other studies throughout the tropics of Australia (Table 5). Indeed, visual comparison of the historical aerial photographs showed that there was also a general increase in cover throughout our study region (data not shown). This is in agreement with the findings of Johansen and Phinn (2005), who reported increased woody vegetation cover in the Wet Tropics Bioregion inferred from Landsat TM/ETM+ imagery from 1988 to 1999, and with reports of savanna thickening in other parts of Australia (Burrows et al. 2002; Fensham and Fairfax 2003; Lehmann et al. 2008) and worldwide (Singh and Joshi 1979; Cabral et al. 2003; Britz and Ward 2007; Wigley et al. 2010).

**Table 5:** Summary of literature on rain forest expansion in Australia

Location	Reference
Kakadu, Northern Territory	Banfai and Bowman (2005, 2006, 2007); Bowman and Dingle (2006)
Litchfield National Park, Northern Territory	Bowman et al. (2001)
Gulf of Carpentaria, Northern Territory	Brook and Bowman (2006)
Cape York Peninsula, Queensland	Russell–Smith et al. (2004b)
Atherton, Queensland	Harrington and Sanderson (1994)
Herberton, Queensland	Unwin (1983, 1989)
Kirrama, Queensland	Harrington and Sanderson (1994)
Mossman, Queensland	Lawson et al. (2007)
Mt. Spurgeon, Queensland	Harrington and Sanderson (1994)

Unwin (1989) measured rain forest boundary dynamics on a transect in the Herberton highlands (near the Clohesy study site in the current study) over a 10-year study period, and estimated that rain forest was expanding at 1 m year^–1^, which is similar to our upper estimate (45 m in 53 years, equivalent to 0.8 m year^–1^). However, our results suggest that on a broader landscape scale, rain forest expansion typically occurs at a much slower rate (Fig. 2; Table 3).

Our spatially explicit modeling approach showed that expansion occurred indiscriminately on all geologies and at all elevations, although the rate and amount of expansion varied among the five sites. The reasons for the different extent of rain forest expansion between the five sites could not be explained by the set of environmental variables used in our analyses. Past logging of tall eucalypt forest and rain forest (Crome et al. 1992) does not appear to have influenced rain forest expansion, given that rain forest expansion was found on all sites regardless of logging history. For instance, the Mt. Carbine site has not been logged but still exhibited rain forest expansion. Nonetheless, rain forest was found to exhibit a higher probability of expansion into tall eucalypt forest (Fig. 4) than into more open forest and woodland types. This was expected, as the environment in tall eucalypt forest is probably more amenable to rain forest regeneration (Unwin 1989). That rain forest expanded into both tall open forest and other woodland types, albeit more slowly than into woodland types, bolsters our conclusion that this process occurs indiscriminately throughout the study area.

Our statistical modeling showed that the only significant correlate of rain forest expansion was distance to preexisting rain forest. This finding is concordant with Banfai et al. (2007) who demonstrated that monsoonal rain forest expansion in Kakadu was most strongly correlated with distance to preexisting rain forest. Contrastingly, field surveys undertaken by Russell–Smith et al. (2004a) suggests that rain forest in the Iron Range region of Cape York also expands via a process of “nucleation” around focal trees in the savanna leading to an eventual rain forest “irruption” via coalescence of nuclei. It is possible that successional processes such as nucleation and irruption are difficult to quantify using GIS methods, particularly when such successional processes are in the early stages.

Our findings are broadly consistent with a diversity of localized studies in northern Australia (Table 5). Ash (1988) argued that rain forest boundaries in the Wet Tropics are strongly controlled by environmental factors, such as geological disjunctions and precipitation gradients, which results in their stability. Topography can also provide “fire shadows” to protect rainforest from frequent fires that occur in eucalypt savannas (Webb 1968; Bowman 2000). Russell–Smith et al. (2004b) found rain forest expansion in the Iron Range on eastern Cape York Peninsula across all geologies sampled, but they also detected a higher probability of rain forest expansion on more fertile geologies. Harrington and Sanderson (1994) reported rain forest expansion in the Mt. Spurgeon area (part of the Mount Carbine region in the current study) using visual interpretation and manually delineating vegetation types from aerial photography taken in the 1940s to the 1990s.

Harrington and Sanderson (1994) suggested that the expansion of rain forest into tall open forest is a threatening process to native mammals such as the Yellow-bellied Glider (*Petaurus australis*) and Brush-tailed Bettong (*Bettongia tropica*) that are restricted to tall eucalypt forest habitats. Their work sparked concern for the fate of these forests and led to calls for managers to use fire to limit rain forest expansion. However, our projections of rain forest expansion show that these tall open forests will largely remain intact within the next century, and will still persist within the next 2000 years (Fig. 5 and 6). The initial steep rate of rain forest engulfment in the first 250 years (Fig. 5 and 6) represents the infilling of embayments of tall open forest existing near the rain forest margins. It is important to note that our analysis was based on the very unlikely scenario that the region would remain undisturbed by landscape fires, and therefore represents an exaggeration of the actual trajectory of rain forest expansion. There is no doubt that some combination of tropical cyclones, droughts, and landscape fires within the next 2000 years will push back at least some rain forest margins, and stimulate large-scale natural regeneration of tall open forest that is generally thought to depend on disturbance for regeneration (Ashton 1981, Adam 1992). Further, even if all the tall open forest understoreys currently have a rain forest understorey, the decline of the overstorey eucalypts, particularly those dominated by *E. grandis*, may take another couple of centuries, given the inherent longevity of tall open forest eucalypts (e.g., 500 years in *E. regnans* [Wood et al. 2010], a eucalypt species similar to *E. grandis* in habit and regenerative strategies). Moreover, it is possible that *E. grandis* forests are unstable ecotonal states that will shift spatially as the rain forest expands outwards (Warman and Moles 2009).

It has been suggested that European colonization and related pastoral activities may have altered the fire regimes previously affected by Aborigines and lightning strikes (Unwin 1983, 1989; Ash 1988). If fire suppression since European colonization was a key driver of rain forest expansion, we would expect a clear signal of expansion from fire-protected areas near drainage systems, or topographically protected areas (e.g., Brook and Bowman 2006), rather than the trend of indiscriminate expansion. Further, the palynological record shows that Aboriginal landscape burning was unable to stop the climate-driven expansion of rain forest at the commencement of the Holocene (Haberle 2005), supporting our view that changed fire regimes are not the explanation for the expansion of rainforest.

Several global drivers of vegetation change have been proposed in the recent literature, which include increased temperature, rainfall, atmospheric nitrogen deposition, and atmospheric CO_2_ concentrations. In savannas, rainfall, rather than temperature, is more likely to influence tree cover, particularly during extreme events such as prolonged droughts (Fensham et al. 2005). Likewise, humid tropical forests depend on abundant and regular water supply and drying trends can result in forest retraction (Behling 2002; Pennington et al. 2004; Silva et al. 2009). Mean annual rainfall for northeastern Queensland in the last decade fell by more than 2% compared to the previous 30 years (Queensland Government 2011), suggesting that a wetting trend is not responsible for the rain forest expansion.

Atmospheric nitrogen deposition is another candidate driver of vegetation change (Pearson and Steward 1993). However, the magnitude of atmospheric nitrogen deposition, and the effects, if any, on the terrestrial vegetation has not been studied in Australia and there is no regional source for this pollution, unlike many regions in the northern hemisphere. Also, atmospheric nitrogen deposition has been found to have a more significant effect on species composition within ecosystems, rather than large increases in biomass (Matson et al. 2002; Bobbink et al. 2010).

With the exclusion of rainfall, temperature effects, and nitrogen deposition, the most parsimonious explanation for the indiscriminate rain forest expansion in the current study is the increase in atmospheric CO_2_, consistent with earlier explanations of landscape-scale rain forest expansion in the Australian monsoon tropics (Banfai and Bowman 2005, 2006, 2007) and elsewhere (Wigley et al. 2010). Bowman et al. (2010) suggest that rain forest expansion is a signal of global environmental change that is so strong that it is overwhelming any retardant effect fire might have on rain forest. The implications of this vegetational shift from flammable savanna and eucalypt forest to rainforest are significant not only at a local scale for biodiversity and management, but may constitute an important carbon cycle feedback at a global scale. Continued rain forest expansion in tropical regions worldwide could possibly instigate a cascade of feedbacks resulting in further land cover changes due to changes in carbon sequestration, albedo, evapotranspiration, fire incidence, cloud nucleation among others (Wigley et al. 2010), and has the potential to significantly alter the earth system within a relatively short time frame.
